# Production of secondary metabolites using tissue culture-based biotechnological applications

**DOI:** 10.3389/fpls.2023.1132555

**Published:** 2023-06-29

**Authors:** Ibrahim Ilker Ozyigit, Ilhan Dogan, Asli Hocaoglu-Ozyigit, Bestenur Yalcin, Aysegul Erdogan, Ibrahim Ertugrul Yalcin, Evren Cabi, Yilmaz Kaya

**Affiliations:** ^1^ Department of Biology, Faculty of Science, Marmara University, Istanbul, Türkiye; ^2^ Department of Medical Services and Techniques, Akyazi Vocational School of Health Services, Sakarya University of Applied Science, Sakarya, Türkiye; ^3^ Biology Program, Institute of Pure and Applied Sciences, Tekirdag Namık Kemal University, Tekirdag, Türkiye; ^4^ Department of Medical Laboratory Techniques, Vocational School of Health Services, Bahcesehir University, Istanbul, Türkiye; ^5^ Application and Research Centre for Testing and Analysis, EGE MATAL, Chromatography and Spectroscopy Laboratory, Ege University, Izmir, Türkiye; ^6^ Department of Civil Engineering, Faculty of Engineering and Natural Sciences, Bahcesehir University, Istanbul, Türkiye; ^7^ Department of Biology, Faculty of Arts and Sciences, Tekirdag Namık Kemal University, Tekirdag, Türkiye; ^8^ Department of Biology, Faculty of Science, Kyrgyz-Turkish Manas University, Bishkek, Kyrgyzstan; ^9^ Department of Agricultural Biotechnology, Faculty of Agriculture, Ondokuz Mayis University, Samsun, Türkiye

**Keywords:** Tissue culture, gene transfer, bioreactor, hairy root, bioengineering

## Abstract

Plants are the sources of many bioactive secondary metabolites which are present in plant organs including leaves, stems, roots, and flowers. Although they provide advantages to the plants in many cases, they are not necessary for metabolisms related to growth, development, and reproduction. They are specific to plant species and are precursor substances, which can be modified for generations of various compounds in different plant species. Secondary metabolites are used in many industries, including dye, food processing and cosmetic industries, and in agricultural control as well as being used as pharmaceutical raw materials by humans. For this reason, the demand is high; therefore, they are needed to be obtained in large volumes and the large productions can be achieved using biotechnological methods in addition to production, being done with classical methods. For this, plant biotechnology can be put in action through using different methods. The most important of these methods include tissue culture and gene transfer. The genetically modified plants are agriculturally more productive and are commercially more effective and are valuable tools for industrial and medical purposes as well as being the sources of many secondary metabolites of therapeutic importance. With plant tissue culture applications, which are also the first step in obtaining transgenic plants with having desirable characteristics, it is possible to produce specific secondary metabolites in large-scale through using whole plants or using specific tissues of these plants in laboratory conditions. Currently, many studies are going on this subject, and some of them receiving attention are found to be taken place in plant biotechnology and having promising applications. In this work, particularly benefits of secondary metabolites, and their productions through tissue culture-based biotechnological applications are discussed using literature with presence of current studies.

## 1 Introduction

Plants produce a large number of secondary metabolites that do not appear to be involved in primary biological activities. Although secondary metabolites are not essential for the continuity of the plant’s vital functions, they provide great benefits in optimizing plant growth, adapting to changing environmental conditions and protecting the plant from environmental damage (Li et al., 2021; Seker and Erdogan, 2023). This shows the great structural diversity of secondary metabolites in different plants as well as in their different parts. While some of these secondary compounds can be very selectively produced in floral tissues to attract pollinators, some can be synthesized in roots or in leaf tissues for defense purposes (Ritmejeryte et al., 2020; Jasuja, 2022). Some derivatives of these secondary metabolites are vital for plant growth as they form hormones such as auxins, brassinosteroids, gibberellins, and strigolactones (Mihalache et al., 2015; Chopra and Samuel, 2020; Sathyanathan and Varadarajan, 2021; Waratadar et al., 2021; Ozbilen et al., 2022).

Previous researches have focused on the effects of growth conditions and environmental stressors (such as drought, salinity, extreme temperatures, and high light intensity) on secondary metabolite synthesis. Jasmonic acid and methyl jasmonate are critical regulators of secondary metabolite synthesis. For example, in presence of pathogens or other stress conditions, the concentrations of jasmonic acid in plant tissues increase (Gutierrez-Gamboa et al., 2021; Nabi et al., 2021; Kuru et al., 2023). Besides jasmonic acid, cytokinins and ethylene are effective on the regulation of secondary metabolites. Cytokinin affects cell division and plant growth at almost all stages especially tuber formation and lateral bud elongation in rice (Zhang et al., 2010; Kaur et al., 2021). On the other hand, ethylene induces the synthesis of some important secondary metabolites such as anthocyanins in most fruits (Ni et al., 2021). Ethylene also induces the release of cell wall–modifying enzymes such as pectate lyase, pectin methyl esterase and polygalacturonase, and affects the regulation of stress-responsive genes (Uluisik et al., 2016; Iqbal et al., 2017; Wang et al., 2019).

Secondary metabolites are often categorized into three major classes based on their biosynthesis pathways: phenolics, terpenes, and alkaloids (Ozyigit, 2008; Eguchi et al., 2019; Jain et al., 2019; Movahedi et al., 2021; Diyabalanage, 2022). These phytochemicals exhibit enormous chemical and biological diversity, are species- and organ-specific, and are produced in response to a variety of biotic and abiotic stimuli (Sharma et al., 2022). As being one of the major classes of plant secondary metabolites, phenolics are common in all higher plants and are involved in lignin biosynthesis (Ozyigit et al., 2007a; Ozyigit, 2008; Khalofah et al., 2021; Bahrami-Rad et al., 2022; Magray et al., 2023), and pigmentation (Pospisil et al., 2021). Among secondary metabolites, polyphenols are present in all plant species and acts in chemical defense systems against the deleterious effects of UV radiations, pathogens, and oxidative stress (Erb and Kliebenstein, 2020; Pradhan et al., 2020). Phenolics are an essential group of active compounds in phytonutrients and complementary medical systems. Modified polyphenols are also known for having various biological roles including antibacterial, and antioxidant activities (Cano-Avendaño et al., 2021). It has also been reported that plant phenolics have potential to be used as antiviral agents against influenza viruses (Wani et al., 2021) and human coronavirus (Aati et al., 2022). A recent study implied that virus-host cell interactions can be disrupted by phenolic secondary metabolites through interfering enzymatic reactions; thus, the severity of viral diseases is reduced (Arimboor, 2021).

Today, there are more than 25,000 terpenoids proven to exist in plants. Terpenes, also known as isoprenoids or terpeneoids, are organic compounds that can be classified as monoterpenes (10 Cs), sesquiterpenes (15 Cs), diterpenes (20 Cs), triterpenes (30 Cs) and other terpenes according to the number of isoprene units (hydrocarbons containing 5 carbon). The large number of different terpene synthases, found in plants, are the primary cause of the molecular diversity of terpenoids in plants; hence, some terpene synthase enzymes can produce different products from the same substrate. Moreover, it has been reported that terpenes have antimicrobial properties against antibiotic-resistant bacteria through inhibiting protein or DNA synthesis or through disrupting the structure of the cell membrane that cause cell breakdown (Masyita et al., 2022). On the other hand, other compounds including alkaloids are rarely existed and more specific to some plant genera and species (Yang and Stöckigt, 2010; Choi et al., 2022; Zhao et al., 2022). Alkaloids are the important part of the defense mechanisms in various plant species and are getting attention worldwide due to their potential to be used in cancer treatment and other therapeutic purposes (Srivastava and Tiwari, 2022). These low molecular weight organic compounds contain alkyl substituted peptide rings; therefore, they can form chemical interactions with many other molecules. Alkaloids have antibacterial, anti-inflammatory and antimicrobial properties (Yan et al., 2021). Alkaloids suppress oncogenesis by modulating some signaling pathways, related to cell division and proliferation as well as metastasis, and due to having these properties, alkaloids have become the focus of many clinical anti-cancer studies (Bello-Martínez et al., 2022). Paclitaxel, vinblastine, vincristine and vitexin are the primary alkaloid-based molecules that are frequently used as anti-cancer molecules in clinical studies (Desam and Al-Rajab, 2022).

The identities of secondary metabolites are used as the basis for chemotaxonomical and chemical ecology studies (Singh, 2016). Compounds with known biological functions in the secondary metabolite class include dyes (shikonin, indigo, etc.), fragrances (lavender, rose, and other essential oils, etc.), spices (mustard oil, capsaicin, vanillin, etc.), stimulants (caffeine, nicotine, ephedrine, etc.), insecticides (nicotine, rotenone, pyrethrin, piperine, etc.), hallucinogens (morphine, scopolamine, cocaine, tetrahydrocannabinol, etc.), some poisons (aconite, coniine, colchicine, cardiac glycosides, strychnine, etc.) and therapeutic drugs (atropine, cardenolide, codeine, quinine, etc.) have been recognized by humans for thousands of years, but today they are the subject of many new studies (Anulika et al., 2016; Alamgir, 2018; Twaij and Hasan, 2022).

Secondary metabolites can be used in industry as raw materials for the production of pharmaceuticals and cosmetics, and as food additives in food industry as well as protecting crops in agriculture. Because of having great importance, there is a wide range of studies on production of secondary metabolites, including gene modification and biosynthesis-related prospects for meeting high value agroecosystem demands. These unique bioactive plant-derived molecules are used as insecticides (laurine, chlorobutanol etc.) (Zhang et al., 2017), hallucinogens (morphine, scopolamine, tetrahydrocannabinol etc.) (Batool et al., 2020), therapeutic agents (codeine, atropine, cardenolide etc.) (Rashid et al., 2021), antioxidants (carsonic acid, rosemary oil etc.) (Sahoo et al., 2022) as well as flavors (capsaicin, vanillin, mustard oils etc.) (Zachariah and Leela, 2018), oils and fragrances (rose and lavender oils, pulegone etc.). Because secondary metabolites coexist in various plant parts such as leaves, flowers, and stems, extraction, and isolation approaches for obtaining a specific targeted molecule from secondary metabolites play an important role. Regarding this, several techniques have been developed for the extraction of secondary metabolites from plant tissues that are also used in the production of high-volume commercial products. A number of techniques developed as an alternative to traditional extraction techniques (maceration, Soxhlet, and steam distillation) where some limiting conditions exist during applications are as follows: Microwave Assisted Extraction (MAE); Ultrasound-Assisted Extraction (UAE); Supercritical Fluid Extraction (SFE); Pulsed-Electric Field extraction (PEFE); and Enzyme-Assisted Extraction (EAE) (Zhang et al., 2018; Getachew et al., 2020). These latter methods, developed for the extraction of secondary metabolites from plants by preserving their biological activities, both increase the commercial value of these bioactive molecules and increase the performance of their use in the medical field.

Today, modern biotechnology shows rapid growth and unlimited potential, and become as a central branch of science regarding with applications in the fields of agriculture, forestry, environment, medicine and pharmacy, military and different industries. Biotechnology covers the studies related with various physiological and biochemical properties of microorganisms, cell and tissue culture operations, the production of some secondary chemicals, proteins, hormones, antibodies, vitamins, antibiotics and vaccines. Based on genetic recombination, modern biotechnology deals with improving plant or animal properties, and developing microorganisms for specific usages, in agricultural and remediation fields through employing genetic engineering methods (Aslan et al., 2021; Ozyigit, 2020; Vitolo, 2021). Plant biotechnology can be defined as “the use of tissue culture and genetic engineering techniques to produce genetically modified plants that exhibit new or improved desirable characteristics” (Agrios, 2005; Bhatia, 2017).

New approaches based on plant tissue culture practices provide provisions as promoting the modification of source easily and of the extraction of secondary compounds in higher qualities and quantities. Tissue culture methods for plants including callus and *in vitro* propagation ensure the production rate of secondary compounds in higher quantities without inhibiting the effects of the atmosphere (Beigmohamadi et al., 2019; Kong et al., 2019; Erdem and Uysal, 2021). Through using recombinant DNA technology, certain genes including *GUS* (a reporter gene), *NPT II* (as a marker gene), *dehE* and *dehD* (herbicide tolerance) can be transferred between species (Mohamed et al., 2016; Kaya et al., 2020; Nandy et al., 2020; Kapusi and Stoger, 2022; Ozyigit et al., 2022). Related with this, recombinant DNA techniques have also been utilized for promoting increases in the yields of some secondary chemicals through modification of the secondary metabolite pathways (Zolfaghari et al., 2020; Sreenikethanam et al., 2022).

As mentioned above, the systems adapted from using tissue culture techniques should be used to produce different compounds as biotechnological products. In order to produce secondary metabolites, the most successful tissue culture techniques for biotechnological applications include using callus culture, hairy root culture, protoplast culture, and micropropagation approaches.

There are very comprehensive and valuable studies pinpointing the importance of plant secondary metabolites in terms of their functions, biological properties, and activities that are changed under the influence of environmental factors, as well as their potential in the medical and economic fields (Pant et al., 2021; Sudheer and Praveen, 2021; Zheng et al., 2021; Mattosinhos et al., 2022).

Especially plant tissue culture methods have a substantial ground in the productions of secondary metabolites from plants that are used in broad range of different industries including agricultural, dye, food processing and cosmetic industries as well as being used as pharmaceutical raw materials by humans in general. In this study, rather than other studies, the emphasis was not on gene transfer systems but on tissue culture systems, and information about secondary metabolites obtained using classical tissue culture was given. It is our hope that this article emphasizing of the importance of plant secondary metabolites in biotechnological studies will be a useful resource, especially for researchers working in the field of tissue culture.

## 2 Secondary metabolites

### 2.1 Phenolic compounds

Phenolic compounds are one of the largest and most complex classes of secondary metabolites produced by plants and they arise via the pentose phosphate, shikimate, and phenylpropanoid pathways in plants. They have been the subject of numerous chemical, biological, agricultural, and medical studies (Laganà et al., 2019; Chiocchio et al., 2021). As chemically defined, phenolic compounds have hydroxylated aromatic rings at the centers where the hydroxy group is being attached directly to the phenyl, substituted phenyl, or other aryl groups (Alara et al., 2021). Phenolic compounds as exhibiting anti-inflammatory, anticancer, and antioxidant activities have been studied for their roles in the treatment of diabetes, neurodegenerative diseases, hypertension, and cancer (Chen and Zhang, 2021; Rahman et al., 2021a; Singh et al., 2021a). These roles may also be associated with their protective properties against oxidative stress and some diseases (Rahman et al., 2021b; Seker and Erdogan, 2023). Besides, simple phenolics have bacteriocidal, antiseptic, and anti-helminthic activities (Bekkar et al., 2021; Patil et al., 2021). It is a large group of compounds containing phenolic acids, phlorotannins, bromophenols, and flavonoids (Murray et al., 2018).

Till now, more than 8,000 structures of phenolic materials from plants including simple ones (i.e., phenolic acids) to highly polymerized ones (i.e., tannins) are identified. Their roles in growth, reproduction, and providing a contribution to plants’ colours as well as involvement in the facilitating of resistance against ultraviolet radiation, pathogens, parasites, and predators are reported. Due to being ever-present in all plant organs, flavonoids are ubiquitously found in the human diet. Phenolics are the compounds found to be widespread in plant foods including fruits, vegetables, cereals, olives, legumes, chocolate, and in beverages including tea, coffee, beer, wine (Saha et al., 2019; Dable-Tupas et al., 2023). Furthermore, organoleptic activity is at least partially attributed to plant food properties (Dai and Mumper, 2010; Yasien et al., 2022). Simple phenolic acids and flavonoids are found in plants with insoluble free, soluble esterified, and insoluble-bound configurations (Gulsunoglu et al., 2019).

Regarding the oxidation state of the central C ring, there are six subgroups of flavonoids, which are flavones, flavonols, flavanones, isoflavones, anthocyanins, and polyphenols, and they are most abundantly found in our diets (Reddy et al., 2020; Kumar et al., 2021). They show a very wide distribution of plants. In higher plants, flavonoids are involved in UV (ultraviolet) filtration, symbiotic nitrogen fixation, and floral pigmentation as well as playing roles in many processes as chemical messengers, physiological regulators, and cell cycle inhibitors (Baskar et al., 2018; Gupta et al., 2021; Liu et al., 2022).

Due to having a range of biological activities (antioxidant, anti-mutagenic, anti-inflammatory, and anti-viral properties), these compounds are considered to be a fundamental source of therapeutic applications (Ginwala et al., 2019). As putative inducers, certain flavonoids including naringenin, luteolin, and quercetin exert effects on PPAR-γ activation and escalate insulin sensitivity (Saini, 2010). Among the flavonoids, quercetin has the effects on relieving symptoms of diseases including high fever, eczema, asthma, and sinusitis (Sreeram et al., 2021; Sangeetha et al., 2022). Epidemiological studies have shown that heart diseases are inversely related to flavonoid intake. In addition, it is known that flavonoids have a preventive effect on the occurrence of the oxidation of low-density lipopolysaccharides and reduce the risk of the formation of atherosclerosis (Ginwala et al., 2019; Li et al., 2020).

Flavonoids provide health benefits with their wide spectrum of effects and are essential being as constituents in a variety of nutraceutical, pharmaceutical, medical, and cosmetic applications. This is due to their free radical scavenging properties being as strong anti-oxidants, along with their capacity to modulate basic cellular enzyme functions (Karak, 2019; Carsono et al., 2022). As a result of *in vivo* and/or *in vitro* research conducted on flavonoids has shown that flavonoids have anti-oxidant, anti-inflammatory, antipyretic, anti-allergic, anti-ulcer, anti-bacterial, anti-cancer, anti-viral, anti-protozoal, anti-platelets, anti-atherogenic activities (Lesnik and Bren, 2021; Mumtaz et al., 2021; Demir et al., 2022; Puangpraphant et al., 2022; Vo et al., 2022).

Tannin derived from the French “Tanin” is used for defining a range of naturally occurring water-soluble polyphenolic compounds (Khanbabaee and Van Ree, 2001). Tannins, which have two subgroups as hydrolyzable and condensed, form a large group among polyphenols. Hydrolyzable ones have a central core of glucose, or another type of polyol esterified with gallic acid (gallotannins), or with hexahydroxydiphenic acid (ellagitannins) (Dai and Mumper, 2010; Mal and Pal, 2022). As polyphenolic secondary metabolites of higher plants, structurally occurrences of tannins are either as galloyl esters and their derivatives, in which galloyl moieties or their derivatives are attached to a variety of polyol-, catechin- and triterpenoid cores, or as oligomeric and polymeric proanthocyanidins that have possessed of different interflavanyl coupling and substitution patterns (condensed tannins) (Fraga-Corral et al., 2021; Rajasekar et al., 2021).

It is known that tannins, as flavonoids, have antioxidant properties with their free radical scavenging effect and are involved in the complex antioxidant defence system by chelation of transition metals and inhibition of prooxidative enzymes (Koleckar et al., 2008; Pizzi, 2021). Tannins are actively used in the preparation of herbal-based medicines. According to studies, herbal tannins are used as astringents (stopping bleeding, constricting vessels) against diarrhea and as an auretic and anti-inflammatory against stomach and duodenal tumors (Brito-Arias, 2007; Fujiki et al., 2012). In addition, anti-tumor, cardioprotective, anti-inflammatory, and antimicrobial activities are defined for tannins (Hossain et al., 2021; Jing et al., 2022; Maugeri et al., 2022).

### 2.2 Terpenes

The synthesis of terpenoids proceeds via using of isoprenoid units (two five-carbon building blocks). Because of having a large number of building blocks, terpenoids are classified as: monoterpenes such as carvone, geraniol, D-limonene, and peril alcohol; diterpenes such as retinol and retinoic acid; triterpenes such as betulinic acid, lupeol, oleanolic acid, and ursolic acid; and tetraterpenes such as α-carotene, β-carotene, lutein, and lycopene (Thoppil and Bishayee, 2011; Ninkuu et al., 2021; Thomas and Pronin, 2021). The terpene synthases are involved in the biosynthesis of terpenes and related to this; they can easily be modified including new catalytic properties through minor changes in their structures. In the synthesis of monoterpenes, the first step is the formation of geranyl carbocation through dephosphorylation, and ionization of geranyl diphosphate (Bergman and Phillips, 2021). The first step of the sesquiterpene synthesis begins with the ionization of farnesyl diphosphate to farnesyl cation. Also, the formation of nerolidyl cation via isomerization can occur from farnesyl cation (Liang et al., 2021; Kirschning et al., 2022). Two routes are known for the synthesis of diterpenes and the main enzymes for synthesis are diterpene synthases. One route includes a class I type enzyme, which catalyzes the reaction via the ionization of diphosphate and the other route includes a class II type enzyme, which catalyzes the reaction via the substrate protonation at the 14,15-double bond of geranyl diphosphate (Veneziani et al., 2017; Liu et al., 2022). The generation of nonsteroidal triterpenoids is facilitated through the conversion of squalene into oxidosqualene and cyclization following the formation of dammarenyl cation. The enzymes, that catalyze the reaction are oxidosqualene cyclases (Singh and Sharma, 2015; Goyal et al., 2022). Many terpenoid compounds display a wide range of pharmaceutical properties and due to having these properties; nowadays, they are now gaining increased interest for their use in clinical practices. As well-known examples, taxol (diterpene) isolated from *Taxus baccata* and artemisinin (sesquiterpene lactone) isolated from *Artemisia annua* can be given for their antineoplastic and antimalarial potential (Croteau et al., 2006; Pollier et al., 2011). The terpenes have activities related to plant interactions, plant defences, and other environmental stresses (Abbas et al., 2017; Ninkuu et al., 2021).

Monoterpenes are a terpene type that consists of two isoprene units and has the molecular formula of C_10_H_16_ (Sundriyal, 2022). Studies by various researchers have also reported that monoterpenes have antiseptic, anti-cancer, antibacterial, and antifungal properties (Scariot et al., 2021; Silva et al., 2021). Various monoterpene types are used in foods as a flavoring and fragrant additive (Li et al., 2021; Wackett, 2021; Ignea et al., 2022), and in agriculture and animal husbandry due to their insecticidal and pesticide effects (Alam et al., 2022; Almadiy et al., 2022; Song et al., 2022).

Sesquiterpenes, the largest class of terpenes, consist of three isoprene units and are represented by the molecular formula of C_15_H_24_ (dos Santos Franciscato et al., 2022). Some of the important known sesquiterpenes are: bisabolol found in *Matricaria recutita* (Herrera et al., 2022); chamazulene found in *Artemisia absinthium* (Mohammed, 2022); farnesol and cumin found in *Vachellia farnesiana*; guaiazulene found in *Cuminum cyminum* (Gilbertson and Koenig, 1981); and dicarabrol found in *Carpesium abrotanoides* L. (Asteraceae) and *Lactarius indigo* (Jie-Wei et al., 2021).

Diterpenes having four isoprene units are shown by the formula of C_20_H_32_ (Somantri et al., 2022). They are widely found in nature and are defined as compounds having various pharmacological activities (Kemboi et al., 2021). For example, it has been reported by various researchers that diterpenes obtained from the plants belonging to the genus *Taxus* are used in the treatment of prostate, ovarian, lung, and breast cancer (Chen et al., 2021; Acquaviva et al., 2022; Tomiotto-Pellissier et al., 2022).

Triterpenes are terpenes having six isoprene units and being shown by the formula C_30_H_48_ (Luo et al., 2021). Triterpenes are produced by all animals, plants, and fungi (Chaudhary, 2022). Examples of this group are squalene (SKU) found in shark liver oil and stigmasterol, oleanan, and ursan found in soybeans, legumes, and nuts. Triterpenes are used in food, cosmetic, and pharmaceutical industry due to their antioxidant, anti-viral, anti-inflammatory, and anti-tumor activities (Sureda et al., 2021; Darshani et al., 2022; Miranda et al., 2022).

Tetraterpenes (Carotenoids) are defined by the formula C_40_H_56_ (da Silveira Vasconcelos et al., 2020; Zia-Ul-Haq, 2021). Examples of tetraterpenes consisting of eight isoprene units are the carotenoids found in peaches, carrots, apricots, spinach, and peppers. Carotenes are tetraterpenes with important biological functions including light capture, antioxidant activity and protection against free radicals, synthesis of plant hormones, and structural components of membranes (Săvescu, 2021; Adil et al., 2022; Seker and Erdogan, 2023). In addition, carotenoids are high-value compounds for the food and pharmaceutical industries and can be synthesized via photosynthetic and non-photosynthetic organisms. Xanthophylls are another group of tetraterpene pigments commonly found in nature (Siziya et al., 2022). Carotenoids function as antioxidants, anti-inflammatory, anti-cancer, anti-diabetic, anti-microbial, and autoinflammatory compounds (Sathasivam and Ki, 2018; Karpiński et al., 2021; Zia-Ul-Haq et al., 2021).

### 2.3 Alkaloids

Some alkaloids containing basic nitrogen atoms are well recognized as biologically active natural compounds in chemistry and medicine. Target-oriented achievements for the synthesis of alkaloids in laboratory conditions can make possible the study and optimization of their biological properties; however, proceeding in their preparations cannot be that much simpler because of the basicity and nucleophilicity of nitrogen, its susceptibility to oxidation, and its ability to alter reaction outcomes in unexpected ways (Parr et al., 2015; Thawabteh et al., 2021). The main key in alkaloid classification is related to the structure of the molecule containing a basic nitrogen atom at any position that does not bear nitrogen in an amide or peptide bond (Bribi, 2018). Some groups of alkaloids also contain bonding properties related to neutral or weak acidity. In addition to carbon, hydrogen, and nitrogen groups, they also contain groups including oxygen, sulphur, and albeit very little, bromine, chlorine, and phosphorus. Compounds such as amino acids, proteins, peptides, nucleic acids, and amines are generally not called alkaloids (Nicolaou and Chen, 2011; Chen et al., 2021). Alkaloids with complex and diverse structures can be classified most commonly and correctly based on their C-N skeleton profiles. Pyrrolidine, pyridine, quinoline, isoquinoline, indole, quinazoline, steroidal, diterpenoid, and other alkaloids are the groups that alkaloids fall into based on the last signature (Archana and Nagadesi, 2022). They can also be produced by a wide variety of organisms such as bacteria, fungi, animals, and plants (especially). Many are toxic to other organisms and have a wide variety of pharmacological activities (Al-Snafi, 2021; Yan et al., 2021; Cano Ortiz et al., 2022). Alkaloids exhibit various activities including toxicity at organismal and cellular levels in herbivores and vertebrates, and in certain bacteria, fungi, and viruses because of having antibacterial, antifungal, and antiviral properties as well as having effects on molecular targets related to mutagenicity or carcinogenicity. Several alkaloids including nicotine and anabasine are useful in controlling insects as insecticides. Many alkaloids with activity on the nervous system are known in animals (Bribi, 2018; Badri et al., 2019; Hussein and El-Anssary, 2019). The most typical example is morphine, a benzylisoquinoline alkaloid formed as a result of the phenol coupling reaction. Alkaloids such as caulerpin, abisindole alkaloid obtained from algae are isolated due to having anti-inflammatory, anti-tumor, and growth regulatory activities (Bai et al., 2021; Haghighi and Ali, 2021; Zhou et al., 2021).

## 3 Secondary metabolite extraction from plant material

Secondary metabolites are distinguished by their ability to accumulate in high concentrations in specific tissues or organs of the plants from which they are synthesized. Secondary metabolites can account for up to 1-3% of a plant’s dry weight (Morris et al., 2021). Having different molecular structures and chemical activities, the unique functions of plant secondary metabolites are more apparent, especially in their pure forms. Plant secondary metabolites are an important starting material for many industrial products and are also valuable for a variety of medicinal products and applications. Therefore, the extraction of these plant secondary metabolites with special biological activity with high efficiency is important both commercially and medically.

Techniques applied for the extraction of secondary metabolites are basically divided into two groups: traditional and untraditional. In traditional extraction techniques such as maceration, Soxhlet, and steam distillation, water or organic solvents are used in terms of employing the extraction power of the solvents as well as heat or mechanical mixing for the extraction. To ovecome the most important restrictions of the traditional methods for example reducing the bioactivity and bioavailability of the target biomolecule, modern techniques such as MAE, UAE, SFE, PEFE and EAE were developed to be used in the extraction of plant secondary metabolites.

### 3.1 Maceration

Being as simple and common, this technique is applied through using of the grounded plant material with a suitable organic solvent (hexane, acetone, methanol, ethanol etc.) together in a reaction vessel (Ashibuogwu et al., 2022). In this method, where the extraction rate can be increased by factors such as heat and mechanical mixing, the extraction process is stopped when the secondary metabolite quantities remaining in the extract and plant material reach equilibrium. The maceration technique, of which works with relatively small volumes and is quite suitable for laboratory-scale extractions, has its own disadvantages and they are: the need for separating of the extract and plant material from each other by a second process (filtration, centrifugation etc.) at the end of the extraction; the need of the time for processing, in which vary from a few hours to several days; the need of a large amount of solvents in the step-applied maceration; and significant losses of secondary metabolites during isolation process after maceration, in which is resulted in low extraction yields (Silva et al., 2021; Pataro et al., 2022; Wela et al., 2022).

### 3.2 Soxhlet extraction

Soxhlet extraction is a technique preferred for the secondary metabolite extraction because of its ease of use. The grounded plant material is placed into a cellulose filled thimble and after following is put into the extraction tube. A suitable solvent is introduced into the extraction flask and reflux is initiated by heat application. The solvent applied gone and condensed into the extraction tube reaches to the plant material in the thimble and descends from there to the following collection chamber. The solvent that is reheated in the chamber passes into the gas phase and then returns to the plant material for a new wash (Khongthaw et al., 2022). The process shows a continuous character with requiring shorter time and taking less solvent consumption compared to maceration (Tzanova et al., 2020). However, it is a significant disadvantage that the extract is kept around the boiling point of the solvent throughout the process (Adewale et al., 2022).

### 3.3 Steam-distillation

This technique is mainly applied for the extraction of volatile plant components such as essential oils, dry or wet plant materials that are dispersed in water (Kshyap et al., 2021). This mixture, being in a container connected to the condenser, is heated, and the resulting steam formed after condenses in the condenser, which is having a two-phase system consisting of extracted essential oil and water in the condensation vessel. Phases are separated using a simple separatory funnel. This technique is suitable for processing large quantities of plant materials, especially for industrial extractions. However, it is not recommended as a suitable technique for the extraction of thermolabile metabolites (Lo et al., 2021).

### 3.4 Microwave-assisted extraction

Microwave-assisted extraction (MAE) is a widely used technique supporting for extracting valuable bioactive molecules (secondary metabolites etc.) from plant materials. MAE is based on the principle that the electromagnetic radiation sent into a polar solvent is absorbed by the substance, and the absorbed energy by the substance increases the intermolecular and intramolecular mobility, resulting in releasing of heat under the “friction force” of the molecules (Delazar et al., 2012). The determinative factors for the efficiency of MAE include the molecular structures of the solvents used (polar or non-polar etc.), the sample/solvent ratio used, the microwave power applied, the time and temperature applied. Although the applied microwave power increases the extraction efficiency, the application of high power may cause overheating of the extraction solution and the subsequent degradation of thermolabile secondary metabolites under the influence of heat. Also, the kind of solvent used in MAE is another important parameter that affects the extraction efficiency. Although the use of water is quite successful due to its ability to absorb microwave energy and provide a homogeneous distribution of the resulting heat, the majority of secondary metabolites derived from plants have very low solubility in aquatic environments. For this reason, various solvent mixtures have been tested by previous studies in order to find a polar and effective solvation environment and their results have been presented (He et al., 2013; Abdelhamid et al., 2019). Besides, numerous studies have also been carried out for the examination of the effectiveness of various reaction parameters in terms of having increased extraction yield using statistical methods (Dang et al., 2018: Poole et al., 2019).

One of the leading advantages of MAE is that the solution system responds very quickly to application, allowing for much greater extraction of total phenolic compounds compared to traditional methods. Microwave energy, being in the frequency range of 300 MHz-1000 GHz, penetrates deeply into the sample, permitting the material to heat up quickly as a whole. Compared to the conditions with long-term and high temperature (110°C) applied in traditional methods, MAE paves the way for shorter-term and higher-efficiency extraction procedures (Magnusson et al., 2017).

### 3.5 Ultrasound-assisted extraction

This technique works based on the fact that ultrasound waves interact with the material, creating changes in the material at the molecular level as well as in physical properties. The ultrasound wave sent on the sample is dispersed in the solvent medium promoting solvent penetration into the cellular matrices, as well as having a disruptive effect on the cell membrane; thereby, significantly increases the secondary metabolite extraction efficiency (Awad et al., 2012). UAE, using microwave frequencies in the range of 20 kHz-200 kHz, stands out as a technique that operates with short processing times and provides to have high extraction efficiencies with very high qualities (Ho et al., 2016). UAE is also a clean technique, not requiring large quantities of solvents in extraction processes. Compared to traditional techniques such as maceration, steam distillation or Soxhlet, MAE provides benefits including simplification of the working procedure, higher efficiency in a shorter time, high purity level in the final product obtained, and low energy consumption (Mushtaq et al., 2020).

### 3.6 Supercritical fluid extraction

Supercritical fluid refers to any substance, found at certain temperatures over the critical level as well as, being found under certain pressure conditions over the critical level (Maliki et al., 2022). The properties of a substance such as density, viscosity, surface tension and diffusibility under supercritical conditions are distinctly different compared to the properties of the same substance stored under standard atmospheric conditions (Radivojac et al., 2021).

Today, the increasing sensitivity for the environmental pollution pushes both industrial production centers and researchers to take different precautions to eliminate pollution sources and to reduce pollution levels. In SFE technique, the most commonly used supercritical fluid is CO_2_ (31.4°C-73.8 bar for CO_2_), but water, ammonia, nitrous oxide and low molecular weight organic compounds (ethane, propane, butane etc.) kept under supercritical conditions can also be used as supercritical fluids (Gumerov et al., 2021; Kessler et al., 2022). The main advantages of SFE technique are that the usage of predominantly non-toxic supercritical fluids, being safer in terms of preventing contamination, allowing selective extraction depending on the supercritical fluid used, and minimizing the oxidative and thermal degradation of the targeted secondary metabolites (Thota et al., 2022). There are also some limitations/disadvantages associated with SFE technique. Among these, additional costs incurred during the generation of supercritical pressure conditions and the necessity of taking additional precautions due to working under high pressure conditions are of importance (Geow et al., 2021).

### 3.7 Pulsed-electric field extraction

PEFE is an unthermal technique for secondary metabolite extraction with minimal processing using plant material. It is an application of repetitive short high voltage pulse (μs-ms) that the applied electric field strengths is in the range of 0.1-3 kV.cm-1 and the energy applied is in the level of 1 - 20 kJ.kg-1. The logical basis of this technique relies on the creation of pores on the cell membranes and thereby increasing the cell membrane permeability that is gained via leaving the viable cells of the sample under the influence of an electric field. Furthermore, the use of this technique, which causes irreversible damage to the sample’s cells, is sometimes referred to as a “pre-extraction process” (Baiano and Del Nobile, 2016).

As a “high capital cost” technology that provides a medium extraction efficiency, PEFE has very short application time and is suitable for working with thermolabile molecules (Sun et al., 2020). However, in order to increase the yield for the targeted secondary metabolites, the process should be applied carefully. For example, prolonging of the application time or increasing of the number of applied electric field pulses may cause an increase in temperature in the sample (Brahim et al., 2021).

Enzyme-Assisted Extraction (EAE) is a simpler and safer extraction technique than traditional extraction techniques, with advantages such as the use of non-toxic solvents and the ability to work in low temperature conditions. The underlying fact of EAE is being involving of the enzymatic degradation of cell membranes and the release of secondary metabolites from the cells in an enzyme-containing extraction medium (Cheng et al., 2015). Another important advantage of this technique is that it allows the selective extraction of secondary metabolites (Marathe et al., 2017). Extractions performed in moderate solvent environments yield significant yield percentages for the targeted secondary metabolite(s) (Wijesinghe and Jeon, 2012). Although EAE is a good alternative to traditional extraction techniques, particularly for highly valuable products, it does have some limitations in terms of applicability, particularly for large volume extractions. As final remarks, the high cost and easily degradable nature of enzymes, difficulty in the isolation of the target product from the final reaction mixture at the end of the process, and the low reproducibility make the technique as choice of a high-cost alternative (Wang et al., 2015; Wang et al., 2019).

## 4 Tissue culture-based biotechnological approaches for obtaining secondary metabolites

### 4.1 Plant tissue cultures

The plant tissue culture applications introduced by Haberlandt (1902) have been used for over 100 years. A media composition widely used in tissue culture protocols with modifications was designed by Murashige and Skoog in, 1962 (Sehgal and Khan, 2020). The model systems established regarding to plant tissue culture applications are often used in research to bring solutions to the various problems related to physiological, biochemical, genetic, and structural conditions of plants (Torres, 2012). Organizations around the world put in use plant tissue culture techniques as well as micropropagation to improve economically important crops widely via transforming different explants and following regenerating them under optimized culture conditions (Chandana et al., 2018; Sehgal and Khan, 2020; Erdem and Uysal, 2021).

Tissue culture approaches mainly include the methods used to obtain and grow plant cells or organs in aseptic conditions using direct and indirect ways, and their usage areas are involved in (1) obtaining molecules having high economic worth such as plant-derived secondary metabolites and recombinant proteins used as biopharmaceuticals, (2) plant reproduction by micropropagation method, (3) conservation of rare or endangered plant species, (4) screening of cells rather than plants for advantageous characters (e.g. herbicide resistance/tolerance), (5) obtaining plants free from pathogens, (6) obtaining hybrid species by interspecies hybridization, (7) obtaining plants having new features when somaclonal variation occurs, (8) production of identical sterile hybrid species, (9) generating haploid plants by anther and microspore culture, (10) cross-pollinating of distantly related species and then application of tissue culturing for creating embryos (embryo rescue), (11) creating chromosome duplication and induction of polyploidy, usually achieved by applications of antimitotic agents such as colchicine or oryzalin (e.g. doubled haploids, tetraploids, and other forms of polyploids), (12) generating new species between cross distantly related species by protoplast fusion and regeneration of the novel hybrids, (13) providing a quick studying way of the molecular basis for physiological, biochemical and reproductive mechanisms in plants (e.g. *in vitro* selection for stress tolerant plants), (14) storage of gene resources, (15) utilization of cell cultures in *in vitro* selection studies, (16) large-scale production of artificial seeds by somatic embryogenesis, (17) carrying out germplasm collections and seed conservation, (18) putting into practices for making automated control of cell growth and rational regulation of metabolite processes in order to have contribution to reduce labor costs, and improvements in productivity and (19) using tissue culture serving as a basic tool for transgenic plant production (Ozyigit, 2012; Mukta et al., 2017; Dagustu, 2018; Huyop et al., 2019; Phillips and Garda, 2019; Secgin and Okumus, 2022).

Totipotency special to plants that are highly useful in biotechnological research provides the ability to regenerate a whole plant from a plant part (Finer and Dhillon, 2016). A basic concept in totipotency follows the regeneration of the whole plant from explants, which are the small parts of the plant prepared by dissection of the plant (body, organ, any tissue, etc.) (Bhatia and Dahiya, 2015). Young plants, seedlings, calli, and somatic embryos are preferred to be used in tissue culture systems (Isah et al., 2017; Secgin and Okumus, 2022). Propagating plants *in vitro* using the tissue culture techniques involve two consecutive steps: the first one is the formation of *in vitro* cell/callus/cell suspension and protoplast culture and the latter one is direct or indirect organogenesis or somatic embryogenesis via in vitro regeneration (Aasim et al., 2019).

**Figure 1:**
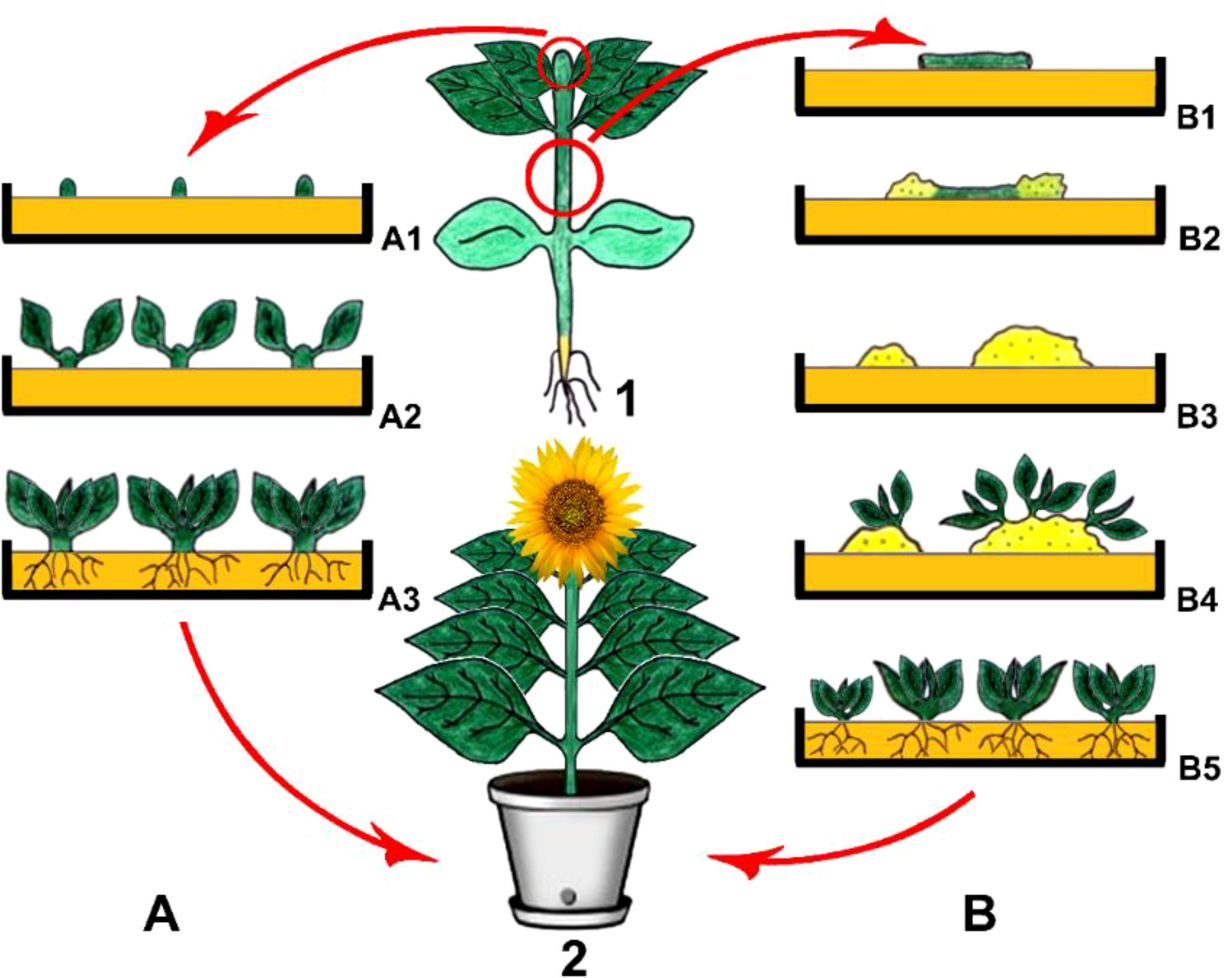
Direct **(A)** and indirect **(B)** organogenesis. 1. Main plant and explant sources, (A1) Isolation of meristematic shoot tips as explants and their culturing, (A2) Direct organogenesis and shoot formation, (A3) Rooting and obtaining young plantlets, (B1) Isolation of hypocotyls as explants and their culturing, (B2) Callus induction, (B3) Young calli, (B4) Indirect organogenesis on callus tissues and shoot formation, (B5) Rooting and obtaining young plantlets, 2 Obtained plant *via* direct and/or indirect organogenesis (Copyrighted illustration from Prof. Ozyigit).

Organogenesis is called the process that involves *in vitro* formation of plant structures including roots, shoots, and leaves through being derived directly from the meristem or indirectly from the callus (**Figure 1**). The plant regeneration process via organogenesis is to be enabled through plant growth regulators that are modified by altering their concentrations in nutrient medium to act on is the formation of callus and differentiation of adventitious meristems into organs (Hussain et al., 2012; Pradhan et al., 2022).

**Figure 2:**
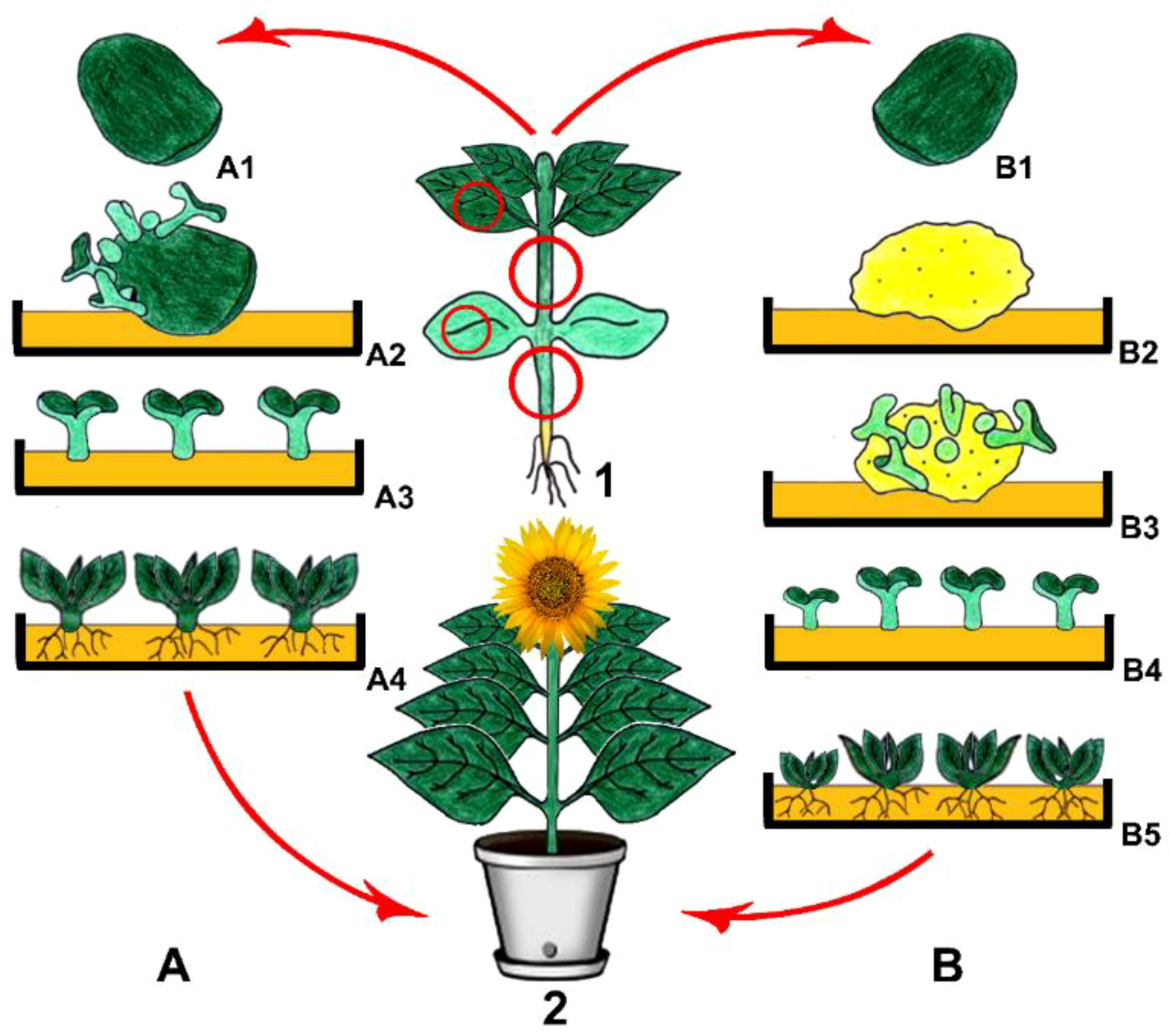
Direct **(A)** and indirect **(B)** somatic embryogenesis. 1. Main plant and explant sources, (A1) Isolation of cotyledones as explants, (A2) Direct somatic embryo development on the explant, (A3) Isolation of somatic embryos and their culturing, (A4) Shoot and root formation and then obtaining young plantlets (B1) Isolation of cotyledonary nodes as explants, (B2) Callus induction, (B3) Indirect somatic embryo development on a young callus, (B4) Isolation of somatic embryos and their culturing, (B5) Shoot and root formation and then obtaining young plantlets, 2. Obtained plant via direct and/or indirect somatic embryogenesis (Copyrighted illustration from Prof. Ozyigit).

Being a non-zygotic embryonic production process, a new plant can be generated through somatic embryogenesis (Secgin and Okumus, 2022). The somatic embryo formation can be carried out either directly from the explant or after the culturing process of the callus (**Figure 2**), involving the first formation of the embryogenic clumps in an auxin-rich medium and the subsequent transfer of the embryogenic clumps into a medium without auxins. Complete embryonic development requirements include adequate delivery of auxin and nitrogen, which are found in the medium, and at the end, whole plant formation is a result of a process involving asexual reproduction (Hussain et al., 2012; Shahzad et al., 2017; Erdem and Uysal, 2021).

The formation of new plants through *in vitro* tissue culture can be defined as a model having mainly six fundamental stages, including: (1) setting up a suitable laboratory environment, (2) choosing a donor plant (identification of plant species and plant parts to be used), (3) determining, preparing and sterilizing of suitable nutrient media for the selected plant species, (4) arranging of callus and cell suspensions, promoting plant regeneration from callus or cell suspensions or regenerating of plants directly from somatic or gamete cells via meristem propagation, organogenesis or somatic embryogenesis, (5) proliferating and extending of the formed shoots, and maturating of somatic embryos, and finally (6) rooting of extended shoots and acclimating of rooting plants (Cui et al., 2019; Joo et al., 2019; Cavallaro et al., 2022).

Productions of important compounds including secondary metabolites via plant tissue culture have also been successfully accomplished. These metabolites are released by plants to provide protection against pathogenic attacks or wounding. Secondary metabolites play important roles in adaptation to stressful conditions (Chandana et al., 2018; Erb and Kliebenstein, 2020). Induction of the biosynthesis of secondary metabolites in plants starts via treatment with any elicitor, which is any stress factor causing triggering of defense responses in plants. (Naik and Al-Khayri, 2016; Khanam et al., 2022). Based on their natures, elicitors are broadly classified into two major categories, being as abiotic (AgNO_3_, CaCl_2_, CdCl_2_, ethanol, and methyl jasmonate, etc.) and biotic (chitosan pectin, chitin, elicitin, yeast extract, and fungal homogenate, etc.) (Kaur and Pati, 2018; Piatczak et al., 2018).

Several elicitation and biotransformation techniques have been utilized to provide a high yield in secondary metabolite production in various plant species (Naik and Al-Khayri, 2016; Halder et al., 2019; Bhaskar et al., 2021; Khanam et al., 2022). Besides, as an alternative tool to increase the growth of cultures by bringing down the cost of the requirements of energy, labor, and space, bioreactors have been started to be used (Shahzad et al., 2017; Abahmane, 2020).

Cell line selection was previously proven to be used in the production of cell lines that can provide great increases in secondary metabolite production (Wawrosch & Zotchev, 2021). *In vitro* production of the berberine in selected cell lines of *Coptis japonica* was reported by Sato and Yamada (1984) with a production of up to 13.2% (DW). *Catharanthus roseus* was utilized by Hall and Yeoman (1987) in the production of high amount of anthocyanin using cell line. Camptothecin formation was realized in suspension cultures of *Ophiorrhiza mungos* through cell line selection, nutrient medium optimization, and jasmonic acid elicitation of 1.12 mg g^-1^ DW compared to 0.06 mg g^-1^ DW in the original cell line (Deepthi and Satheeshkumar, 2017). Recently, Heydari et al. (2020) improved the production of some phenolic acids (rosmarinic, salvianolic-B, ferulic, and cinnamic) in the cell suspension cultures of Woodland Sage through attaining high-yielding cell lines and carboxyl functionalized multi-walled carbon nanotubes elicitation. To provide the needs required by the pharmaceutical industry, attempts have been done in terms of realization of increase in the secondary metabolite production (Hussain et al., 2012).

As mentioned above, it is possible to produce only the relevant plant part for the production of secondary compounds (Mishra, 2015). These strategies include gene cloning and repeated selection of high-yielding strains from heterogeneous cell populations using plant tissue culture techniques such as clonal micropropagation, callus, hairy root, and protoplast cultures (Solle et al., 2016). Several secondary compounds, being produced by using tissue culture techniques from various explant sources, are identified as follows: phenolics including caffeic acid, rosmarinic acid and rosmarinic acid hexoside, salvianolic acid, salvianolic acid K, salvianolic acid F isomer I, salvianolic acid F isomer II, caffeic acid derivative I, caffeic acid derivative II, and methyl rosmarinate from the leaves and shoots of *Salvia bulleyana* (Wojciechowska et al., 2020); iridoid glycosides (aucubin, harpagide, harpagoside) and phenylethanoid glycosides (verbascoside and isoverbascoside) from the seeds, leaves and shoots of *Rehmannia elata* (Piatczak et al., 2019); podophyllotoxin-related compounds (6-methoxy-podophyllotoxin, podophyllotoxin and deoxypodophyllotoxin) from the hypocotyls of *Linum flavum* (Renouard et al., 2018); psoralen, daidzein and genistein bioactive compounds from the cotyledon callus cultures of *Cullen corylifolium* (Singh et al., 2020); triterpenoids (madecassoside, asiaticoside, madecassic acid, and asiatic acid) from the petioles and leaves of *Centella asiatica* (Baek et al., 2020); crocin, pircorcrocin, safronal from the corms of *Crocus sativus* (Ahamed, 2019); phenolic acids (Caffeic acid, Syringic acid, p-Coumaric acid, ferulic acid, Salicylic acid) and flavonoids (rutin, Myricetin and Kaempferol) from the nodes, internodes and leaves of *Sphagneticola calendulacea* (Kundu et al., 2018); meroterpene bakuchiol from the cotyledone-derived callus, seeds, leaves, internodes and roots of *Psoralea drupacea* (Lystvan et al., 2010); tryptophan-derived quinoline alkaloid camptothecin from the shoots and leaves of *Ophiorrhiza alata* (Ya-ut et al., 2011); monoterpene-derived indole alkaloid camptothecin from the radicle-derived roots of *Pyrenacantha volubilis* (Hima et al., 2019); and phenolic acids (Rosmarinic acid, Caffeic acid, Lithospermic acid, Chlorogenic acid, Cinnamic acid) from the leaves and shoots of *Mentha spicata* (Yousefian et al., 2020); flavonoids, phenylpropanoids, alkaloids, fatty acids and aromatic glycosides from callus and suspension cultures of *Carthamus tinctorius* (Liu et al., 2021); epigallocatechin and chlorogenic acid from callus and suspension cultures of *Oryza sativa* (El-Beltagi et al., 2022) and phenolics, flavonoids, tannins and essential oils from nodal segments of *Artemisia arborescens* (Riahi et al., 2022).

#### 4.1.1 Clonal micropropagation

**Figure 3:**
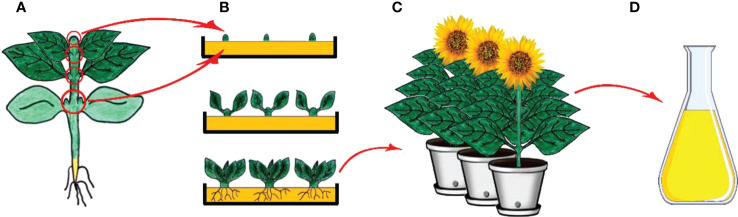
Clonal micropropagation. **(A)** Main plant and meristem derived (shoot tips, leaf nodes and cotyledonary nodes) explants **(B)** Isolation of explants, and *in vitro* direct organogenesis and obtaining clone plants, **(C)** Obtained clone plants, **(D)** Isolation of secondary metabolites from clone plants (Copyrighted illustration from Prof. Ozyigit).

Micropropagation is the process involving the generation of plants from vegetative parts or seeds of plants through growth and multiplication by applying various plant tissue culture techniques and is executed in aseptic and favorable conditions on growth media (**Figure 3**) (Zhou and Wu, 2006; Sidhu, 2011). Micropropagation also presents an advantage as being an effective way of regenerating tissues of genetically transformed material (Bravo-Ruiz et al., 2022; Ozyigit et al., 2022). The regeneration of a plant through the process involves isolation of a plant part (leaf, bud, meristem, etc.) under aseptic conditions and following usage of the plant part isolated via application of a different source of hormone and media regime (Ozyigit and Gozukirmizi, 2008; Sehgal and Khan, 2020). Micropropagation has been shown to be faster and less expensive than traditional cell and tissue culture methods, and it can be used to produce true-type plant material if meristem culture is used (Ozyigit, 2009; Ozyigit and Gozukirmizi, 2009; Musacchi and Neri, 2019; Sehgal and Khan, 2020; Eisa et al., 2022).

The traditional system for agricultural practices allows for clonal propagation of plants, but reproduction rates remain relatively low, which explains why it may take many years for plant tissue culture methods to enter agricultural applications (García-Gonzáles et al., 2010). Through the application of a tissue culture method developed for living plant species, rapid propagations can be effectively achieved, even for the plant species having low multiplication rates (Aslam et al., 2020). As well, the land requirement for the growth of plant species via micropropagation is significantly smaller (Huyop et al., 2019). In conventional cultivation, due to unsuitable climatic conditions or taking a long time, the desired growth and reproduction or germination or flower and seed production by many plant species are insufficient in the field. Micropropagation provides applications to be realized allowing a regular supply of plants using minimum space and time (Prakash and Van Staden, 2007). The main advantages of *in vitro* micropropagation of plants can be listed as (1) Multiplication of plants at high rates in a short time and a small space, (2) Providing plant production all year round without being affected by regional or seasonal variations, (3) Obtaining of clones having desired characteristics, (4) Productions of genetically engineered plants that are newly created or improved, (5) Enabling of cryopreservation of genetic materials, (6) Using of it for growing of virus-free plants such as potato, banana, apple, and papaya, (7) Rapid and large-scale propagations of plants which are endangered or medicinal or economically important and enhancing of production of plant derivatives in high rates (Sidhu, 2011; Sehgal and Khan, 2020). The important point here is to have new plants in large numbers that are able to produce secondary metabolites naturally using micropropagation.

Plants, especially medicinal plants, have also been used in traditional medicine for years; therefore, they are subjects of ongoing research for their potential biotechnological uses due to having chemical properties in medicine and pharmacy (Veraplakorn, 2016; Debnath and Goyali, 2020; Karahan et al., 2020). The aim of using this approach being apart from other biotechnological approaches is not to obtain large-scale secondary compounds but rather to obtain large numbers of clonal plants in a short time from a selected mother plant and isolation of these chemicals from these clonal plants (**Figure 3**).

#### 4.1.2 Callus cultures

**Figure 4:**
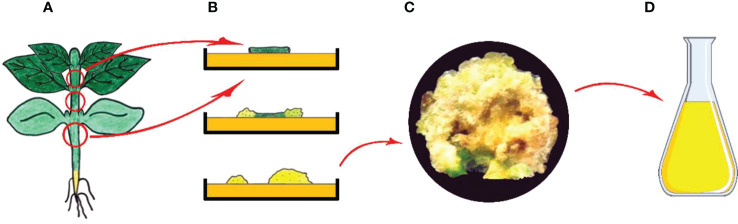
Callus induction and secondary metabolite production from callus cultures. **(A)** Main plant and explant sources, **(B)** Isolation of explant, and *in vitro* callus induction, **(C)** Obtained callus and **(D)** Isolation of secondary metabolites from callus (Copyrighted illustration from Prof. Ozyigit).

As known, a plant cell in culture retains totipotency function; hence, has the ability to produce the substances found in the parent plant (Feher, 2019). In tissue culture practices, callus is formed as a result of unorganized growth seen following the wound healing process along with initiation of cell division, which is started on the surface of freshly dissected explant after transferring it into growth-promoting conditions (Neumann et al., 2009; Filová, 2014). Callus produced via dedifferentiation is plant tissue that predominantly contains an unorganized mass of parenchymatic cells (**Figure 4**) (Ozyigit et al., 2007b).

Callus culture formations have been successfully initiated in a wide variety of dicot and monocot species. Callus formation can be induced using explants from stems, anthers, fruits, apical regions, leaves, roots, flowers, or seeds (Patra et al., 2020). Vascular cambia, storage organs, pericycle of roots, endosperm, cotyledons, leaf mesophyll, and pro vascular tissue are the tissues used for callus production under *in vitro* culture conditions via induction (Torres, 2012; Patra et al., 2020; Ozbilen et al., 2022). Although sometimes seen that liquid medium is used, the solid medium is generally used for callus growth. There are several disadvantages to using solid cultures that make liquid cultures preferable, at least in some cases (Bridgen et al., 2018; Efferth, 2019). For instance, only part of a callus proliferates on the surface of the solid medium; hence, various factors including gradients in nutrients, the exchange of gases, and toxic waste products existing between the callus and the solid medium cause disparity in raising callus. Besides, gravity and variations in light intensity are the parameters that affect the proliferation of the callus by causing polarization. Also, a problem raising with callus growth in the solid medium is associated with limitations in certain directions by the medium or walls of the glassware. Finally, transferring callus grown in a solid medium to a liquid medium cannot be realized without some distortions occurring in the tissue. Solid cultures are still preferred as the method chosen for routine maintenance of callus formation, even though having drawbacks (Torres, 2012; Patra et al., 2020). The media supplemented with relatively high auxin concentrations or a combination of auxin and cytokinin provide *in vitro* conditions required for callus formation (Ozyigit et al., 2002; Shekhawat et al., 2021). Callus formed from explants at the initiative is called a primary callus or callus induction. And then, secondary callus formation starts from the primary callus (Phillips and Garda, 2019; Patra et al., 2020). Callus cultures prepared can be found as embryogenic callus or non-embryogenic callus. Differentiated embryonic competent cells found in embryogenic callus are capable of regenerating whole plants through the somatic embryo development process. Non-embryogenic callus cultures that retain homogenous clumps of dedifferentiated cells are utilized for the production of secondary metabolites (Filová, 2014).

The effective long-term maintenance of callus culture on the same medium cannot be provided due to the occurrence of cell losses causing a reduction in cell division and secondary metabolite production. Therefore, subculturing practice for callus should be regularly repeated over a period of 4-5 weeks (Patra et al., 2020; Wahyuni et al., 2020). Storage products accumulated within resting cells appear to be gradually lost during dedifferentiation. After the formation of new meristematic cells in the tissue, undifferentiated parenchymatous cells without any structural order are developed (Filová, 2014). The natural photosynthetic capacity in most plant cultures is lost as a result of the dedifferentiation process occurring. Consequences of this situation have probably arisen as variations occurring in the culture of callus tissue and the donor plant that have different metabolic profiles (Bhatia and Dahiya, 2015).

After undergoing growth resulting in the formation of a typical unorganized callus, the re-appearance of some kinds of specialized cells can be again seen in the following time of development. Such differentiation can arise randomly but may occur as being taking part of centers of morphogenesis that direct the formation of organs including roots, shoots, and embryos. Unorganized cultures are used for the *de novo* production of plants, often being known as plant regeneration (Filová, 2014). The first step in considering callus cultures to be used in the production of plant secondary metabolites by cell culture is to ensure that they are stable and optimized. For instance, preparing the liquid suspension cultures to be used as inocula. Many previous studies addressed the use of cell suspension cultures for the production of secondary metabolites and considered this technology as a way to overcome problems related to product quantity and quality of whole plants due to the effects of different environmental factors (Rao and Ravishankar, 2002; Yamamoto et al., 2002; Zhang et al., 2002; Filová, 2014).

In addition, some active ingredients and secondary compounds have successfully been produced by application of tissue culture approach using intact plants (**Figure 4**). The examples are as listed: phenolic molecules, including apigenin, p-coumaric acid, genistein, luteolin, rutin hydrate, trans-ferulic acid, salicylic acid and naringenin from *Coryphantha macromeris* (Karakas and Bozat, 2020); medicinally vital phenolic and flavonoid compounds, including apigenin, caffeic acid, catechin, gallic acid, hederagenin, myricetin, kaempherol, isorhamnetin, nahagenin, ursolic acid, betulinic acid from *Fagonia indica* (Khan et al., 2019); p-coumaric acid, hesperidin, cafeic acid, rosmarinic acid from *Rosmarinus officinalis* (Coskun et al., 2019); phenolics, including gallic acid, chlorogenic acid, caffeic acid, rutin, myricetin, quercetin, vanillic acid, luteolin and iso-rhamnetin, from *Lycium barbarum* (Karakas, 2020); gingerol, shogaol, and zingerone from Zingiber officinale (Arijanti and Suryaningsih, 2019); indole alkaloids, including echitamine, acetylechitamine, tubotaiwine and picrinine from *Alstonia scholaris* (Jeet et al., 2020); crocin from *Crocus sativus* (Moradi et al., 2018); anticancer alkaloids (vincristine and vinblastine) from *Catharanthus roseus* (Mekky et al., 2018); phenylethanoid (salidroside, tyrosol), phenylpropanoid (rosavin and rosarin) and phenolic acids (p-coumaric acid, gallic acid, and cinnamic acid) from *Rhodiola imbricata* (Rattan et al., 2020); eugenol and ursolic acid from *Ocimum tenuiflorum* (Sharan et al., 2018); bioactive compounds, including 1,2-benzenedicarboxylic acid (phthalic acid), 3,7,11,15-tetramethyl-2-hexadecen-1-ol, 2-hexadecen-1-ol-3,7,11,15-tetrametil, hexadecanoic acid methyl ester (methyl palmitate), n-hexadecanoic acid (palmitic acid), 9,12-octadecadienoic acid methyl ester, 9,12,15-octadecatrienoic acid methyl ester, phytol, octadecanoic acid methyl ester (methyl stearate), 9,12,15-octadecatrienoic acid (linolenic acid) and squalene from *Mucuna pruriens* (Sweetlin and Daniel, 2020); several different metabolics, including acetamide, propanoic acid, α-thujene, linalool, 5-hydroxymethylfurfural, β-maaliene, epidolichodial, calarene, seychellene, α-curcumene, eremophilene, α-vatirenene, valencene, α-cadinol, ledol, meso-erythritol, α-gurjunene, viridiflorol, (-)-globulol, spirojatamol, dodecanoic acid, patchouli alcohol, jatamansone, xylitol, aristolone, protocatechuic acid, mannose, hexadecanoic acid, p-coumaric acid, talose, α-D-mannopyranose, α-D-galactopyranoside, D-mannitol, myo-inositol, -D-glucopyranoside, D-(+)-trehalose, D-(+)-cellobiose, melibiose, vitamin E, β-sitosterol from *Nardostachys jatamansi* (Bose et al., 2019); identified 11 organic acids, 16 phenolic acids, 8 flavonoids, and 17 metabolites of different classes from *Coryphantha macromeris* (Cabanas-Garcia et al., 2021); phenolic compounds (ferulic acid, isoquercitrin, rutin, quercetin, quercetin-7-O-glucoside and luteolin) from *Hyssopus officinalis* (Babich et al., 2021) and phenylethanoids and steroidal glycosides of the furostanol typefrom *Digitalis lanata* (Tomilova et al., 2022).

#### 4.1.3 Protoplast and suspension cultures

**Figure 5:**
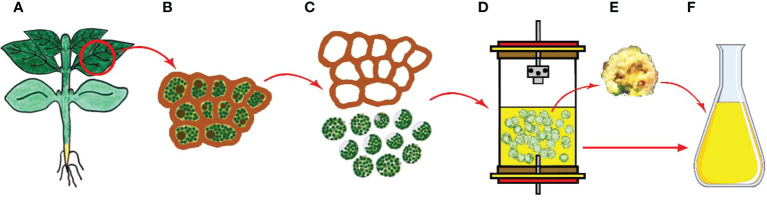
Protoplast cultures and secondary metabolite production. **(A)** Main plant and explant sources, **(B)** Isolation of plant cells from leaf tissue, **(C)** Cell wall removal and obtaining protoplasts, **(D)** Culturing the protoplasts in liquid media, **(E)** Obtaining callus, **(F)** Isolation of secondary metabolites from protoplast derived calli (Copyrighted illustration from Prof. Ozyigit).

Defined as protoplast, the cell has a spherical shape surrounded by a plasma membrane, but without having a cell wall and sensitive to osmotic pressure. The protoplast can regenerate a cell wall and can give rise to callus and shoot, root, or embryo as well as subsequent entire plant formations through the redifferentiation process (El-Sherif, 2018). The usage of protoplasts has wide potential for applications in genetic engineering and crop improvement programs in terms of being capable of making fusion and being able to take up genes. In protoplast creation, mesophyll tissues from leaves are generally used as the preferable source for isolation (**Figure 5**) (Patra et al., 2020).

Suspension culture conditions can be used for protoplasts, as being used for calli. Following the accomplishment of hydrolytic removal of cell wall building materials by using suitable enzymes, the remaining so-called naked cell, known as a protoplast, can be obtained (Kang et al., 2019). Mechanical and/or enzymatic method(s) can be employed for the isolation of protoplasts from cells. In mechanical application, protoplast obtaining is provided by the plasmolysis process resulting in the separation of the cell wall from the cell during shrinkage. The cell walls from the plasmolyzed cells are cut to release the protoplasts without damaging them using a sharp blade (Patra et al., 2020). The process of enzymatic hydrolysis includes the utilization of enzymes in a sequential or mixed fashion. Macerozyme and pectolyase are pectin hydrolyzing enzymes, one of which is used for the separation of the cells. After separation, the cells are washed using cell and protoplast enzyme-free washing (CPW) solution containing only plasmolyticum by gentle centrifugation. After centrifugation, the pellet is kept in the centrifuge tube containing second enzymes (i.e., cellulases, hemicellulases), which are used for hydrolysis of the remaining cell wall materials. CPW application is done for the removal of debris after the release of protoplasts (Silva et al., 2012; Patra et al., 2020). Following protoplast isolation, their viability test is performed and then, the viable protoplasts are cultivated in artificial media at a known concentration (Chadipiralla et al., 2020). An isotonic medium gives better survival ability to these protoplasts, and they stay healthy in such an environment. A wide range of physiological problems related to cell walls is actively studied using protoplasts (i.e., nutrient uptake at the cell wall, mechanisms involved in the cell wall synthesis) (Neumann et al., 2009).

As a suitable source, the best material for protoplast isolation in large quantities is young callus. The procedure for isolation from callus is basically equal to the procedure used for isolation from leaves. One difference is related to the optimal enzyme concentrations (i.e., cellulase) that are used less in the isolation of protoplasts from callus compared to leaf tissues. Being young and showing active growth, cell suspension culture is also proven to be a good source for isolating protoplasts (Torres, 2012). Secondary metabolites can be obtained directly and indirectly from protoplast cultures; however, studies are fewer than those in which secondary compounds are obtained with other methods.

The followings can be given as examples of secondary compounds obtained by protoplast cultures and the plants studied: benzoxazinoids from *Zea mays* (Gao et al., 2019); indole alkaloids from *Catharanthus roseus* (Aoyagi et al., 1998); chitinase, ajmalicine, and 5’-phosphodiesterase from *Wasabia japonica* and *Catharanthus roseus* (Akimoto et al., 1999); scopolamine from *Hyoscyamus muticus* (Oksman-Caldentey and Strauss, 1986); saponins from *Maesa lanceolate* (Lambert and Geelen, 2010); phenolics and flavonoids from *Satureja sahendica* (Tarigholizadeh et al., 2021); and 3-*O*-*p*-coumaroylquinic acid and 3-*O*-feruloylquinic acid from *Bambusa multiplex* (Nomura et al., 2021);

As rapidly dividing homogeneous cell aggregations in suspensions (King, 1984), the suspension cultures can be used in studies related to biochemistry and physiology including growth and metabolism at cellular levels as well as molecular biology and genetic engineering researches. Applications in the industrial level productions of secondary compounds, cell suspension cultures can also be utilized (**Figure 5**) (Loyola-Vargas et al., 2008; Sharifi-Rad et al., 2020).

Isolation of cells from *in vitro* plant materials for the preparation of suspension cultures can be obtained by either mechanical treatment or enzymatic digestion. Other than these, cells for suspension culture preparation can be made from callus induced from any explants. Actually, cell suspension culture can be practically derived from any part of the plant, as in callus cultures. A predictable pattern of growth curve depending upon multiple factors including light, temperature, and aeration can be drawn when a suspension culture is maintained under controlled conditions (Daffalla and Elsheikh, 2018; Patra et al., 2020). A peak with reaching a maximum cell biomass increase for a period of time is seen during incubation. By application of dilution at this point as subculturing, the occurrence of the repeating of the process for the growth and yield is realized (Santos et al., 2019; Wang et al., 2019). Following of entry into the stationary phase occurring as a result of exhaustion of some factors or the accumulation of toxic substances in the medium, a decrease in the viability of cells in the suspension as well as the growth rate for the whole culture is observed. By the addition of an aliquot of the cell suspension into the freshly prepared medium, which has the same composition as the original, new cell suspension can be prepared via the following step (Bhatia and Dahiya, 2015).

There are many options for the method used in the production of appropriate suspension culture. However, using an agitated (50-200 rpm) liquid medium with friable callus added provides the dispersion of the cells in most cases during incubation, after several passages. Mechanical agitation is the cause for most cell suspension cultures to arise from callus cultures. The initiation of suspension cultures can be done using sterile seedlings or embryos (Loyola-Vargas et al., 2008).

After breaking up soft callus using a hand-operated glass homogenizer, the transfer of homogenate to the liquid culture medium is executed (Torres, 2012; Efferth, 2019). Generally, a suspension can be prepared from stationary cultures grown on agar with the aid of a sterile glass rod or by squeezing with a scalpel. In particular, loosely attached cells generated on the opposite side of the agar medium with 2-4 D can be easily scraped off by using a sterile scalpel. An improvement driven by ammonia, being used as a nitrogen source can probably be attributed to the excretion of protons in exchange for its uptake by the cells (Neumann et al., 2009). In ideal conditions, suspension cultures consist of single cells, but rather this is the case rarely seen and appearances as small aggregates formed generally by 20-100 cells are usually observed (Loyola-Vargas et al., 2008). The ideal cell suspension culture is well defined by homogeneity that depends upon both morphologic and biochemical criteria (Torres, 2012). Suspension cultures consisting of a population of cells are nearly homogenous allowing them to be exposed to nutrients easily. Being as useful biological material, cell suspensions offer an opportunity for studying biosynthetic pathways (Shahzad et al., 2017). For the production of beneficial secondary compounds in suspension cultures of different plant species, applications using *rolC* genes have also been carried out for determining their possible stimulatory and inhibitory effects (Bulgakov et al., 2005). Involvement of *rolC* in *Panax ginseng* cells in the plant defence through the induction of related genes has been shown (Kiselev et al., 2006).

All cell culture methods applied for *in vitro* propagation of different plants follow general steps as directed: isolations of the plant cells from the cultured tissue by means of mechanical or enzymatic process; growth and subculturing of batch or continuous propagated suspension cultures; determining of selection and optimization of culture medium conditions for cell suspension culture; making synchronization of suspension cultures; performing physical selection via volume and temperature shock and chemical methods via application of starvation, inhibition, mitotic arrest; growth estimations in suspension cultures through measurements; determining of growth parameters via measurements of cell counting, packed cell volume, cell fresh, and dry weight; assessing cultured cell viability via using phase-contrast microscopy and performing tetrazolium salt reduction, fluorescein diacetate, Evans blue staining assays; and culturing of isolated single cells including involvements of using plating technique, filter paper raft nurse technique, microchamber technique, and scale-up technique (Bhatia and Dahiya, 2015).

Examples of secondary metabolites obtained using suspension cultures can be given as stigmasterol from *Abutilon indicum* (Rao et al., 2021); gymnemic acids from *Gymnema sylvestre* (Mahendran et al., 2021); catechin from *Camellia sinensis* (Ardianto et al., 2020); alkaloids (vincristine, vinblastine, ajmalicine and serpentine) from *Catharanthus roseus* (Mishra et al., 2019); triterpenoids from *Ocimum basilicum* (Pandey et al., 2019); artemisinin from *Artemisia annua* (Mir et al., 2017); plumbagin from *Plumbago europaea* (Beigmohamadi et al., 2019) and *P. zeylanica* (Roy and Bharadvaja, 2019); bacoside from *Bacopa monnieri* (Kharde et al., 2018); hydrolyzable tannin from *Phyllanthus debilis* (Malayaman et al., 2017); triterpenoids from *Euphorbia hirta* (Samkumar et al., 2019); atropine from *Hyoscyamus muticus* (Abdelazeez et al., 2022); triterpenic acids (betulinic acid, oleanolic acid, and ursolic acid) from *Thymus persicus* (Lamiaceae) (Bakhtiar and Mirjalili, 2022); and withanolides (withaferin A and withanolide A) from *Withania coagulans* (Mirjalili and Esmaeili, 2022).

#### 4.1.4 Hairy root cultures

Undifferentiated plant cell cultures are generally found to grow faster compared to roots of higher plants and are considered an alternative to roots of higher plants because of easy handling in the harvesting process. As an alternative method for the production of compounds, the use of plant hairy root cultures is the most promising one (Pence, 2011; Hussain et al., 2012; Filová, 2014).

*Agrobacterium*-based hairy root culture formation for different plant species has been realized using different strains of *Agrobacterium rhizogenes* (a Gram-negative soil bacterium) through causing infection, which comprises of integration of its T-DNA of root inducing plasmid into the host genome (Ozyigit et al., 2013). The roles of rol genes (*rolA*, *rolB*, *rolC*, and *rolD*, which correspond to orf10, orf11, orf12, and orf15) involved in the induction of hairy root formation were first identified in plants infected by the *A. rhizogenes* A4 T-DNA mutants (White et al., 1985). The neoplastic phenotype of root growth arising from *A. rhizogenes* infection is described as showing a high growth rate, high degree of lateral branching, a profusion of root hairs, and lack of geotropism (Hu and Du, 2006; Hakkinen and Oksman-Caldentey, 2018).

**Figure 6:**
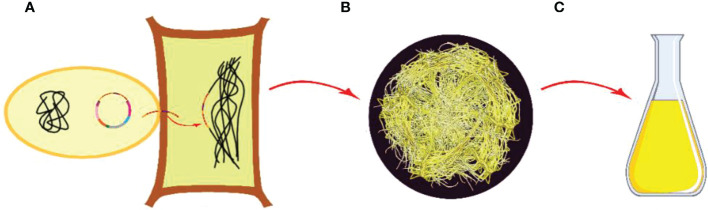
Obtaining hairy roots after gene transfer using *Agrobacterium rhizogenes* and secondary metabolite production from hairy root cultures. **(A)** Gene transfer to plant cell via *A.* rhizogenes, **(B)** *In vitro* induced hairy roots, **(C)** Isolation of secondary metabolites from hairy roots (Copyrighted illustration from Prof. Ozyigit).

Cell and tissue culture systems, which are considered biotechnological tools, have been applied to increase the production of secondary compounds, but their use has brought limited success mainly due to their undifferentiation. (Kaur and Pati, 2018). Genetically engineered root cultures with high stability and high productivity have become a viable choice as a useful biotechnological tool as they allow the use of hairy roots for the production of plant secondary compounds compared to intact plants (**Figure 6**) (Giri and Narasu, 2000; Srivastava and Srivastava, 2007; Pistelli et al., 2010). The intense progress in the development of hairy root technology was gained after having comprehensive knowledge of molecular mechanisms. The critical point in obtaining high yields to produce secondary compounds is the optimization of the nutrient composition (Hu and Du, 2006; Hussain et al., 2012). The main applications using hairy root cultures include biotransformation, production of high-value plant metabolites, phytoremediation, and production of artificial seeds (Georgiev et al., 2012; Guillon et al., 2006; Ozyigit et al., 2021). In addition, the hairy root cultures show distinguishing features as having high genetic stability and being more stable for metabolic production compared to undifferentiated cell cultures (Peebles et al., 2009; Häkkinen et al., 2016). This is mainly due to the high degree of chromosomic stability that hairy roots exhibit (Weber et al., 2008; Weber et al., 2010; Dehghan et al., 2012). Hairy roots have chromosome numbers and karyotypes, which are characteristically the same as that of found in the parent plant. Also, the stability of the growth capacity of hairy roots can be increased without exogenous auxin application. Exposure to growth regulators causes changes in even organized tissues, manifested as modification of chromosome numbers and somaclonal variation (Baíza et al., 1999; Hakkinen and Oksman-Caldentey, 2018).

Concerning the production of plant secondary compounds from hairy root cultures, a number of root nodule research were realized. Many studies have shown that the transformed roots produce high yields in many plant species for *in vitro* secondary compound and artificial seed production (Giri and Narasu, 2000; Erdem and Uysal, 2021). In conjunction with this issue, biosynthesis characteristics of plant secondary compounds in transformed root cultures were analyzed from this perspective (Kuzovkina and Schneider, 2006). The production of plant-based chemicals on large-scales was gained momentum using organized cell cultures after the observation of a strong correlation between secondary compound production and morphological differentiation. Root-shoot co-culturing was an effective way of improving generating tissue-specific secondary compounds using intergeneric co-culture of genetically transformed hairy roots and shooty teratomas (Filová, 2014).

For large-scale production of bioactive substances by employing hairy root-based biotechnology, the key point is the cultivation of bioreactors designed for optimal conditions for having high yields (See section 4.2.) (Georgiev and Weber, 2014). Hairy root cultures are susceptible to shear stress; thereby, bioreactor systems designed for the cultivation of hairy roots show differences from those used for suspension plant cell cultures (Mishra and Ranjan, 2008; Piatczak et al., 2018). The regenerations of the transformed plants by the application of genetic engineering approach with the use of *A. rhizogenes* from hairy roots often show the higher capability of accumulating secondary compounds compared to wild type counterparts and as an alternative and efficient method for obtaining plants for high production of a range of biologically active substances can be used (Korde et al., 2016; Piatczak et al., 2018). On the other hand, for the use of hairy root cultures in the production of bioactive compounds, the construction of a viable bioreactor system with optimum configuration for various physical and chemical parameters including controlled temperature, optimum pH, adequate substrate, salts for nutrition are required. For this purpose, oxygen, product and by-product removal, inoculation size and density, and product recovery should be considered. Also, the Agrobacterial concentration is found to be an important factor in the production of transformed roots (Mishra and Ranjan, 2008).

Although genetically controlled, the nutritional and environmental factors as drawbacks in the biosynthesis of secondary compounds in bioreactors using hairy root cultures can be influential. As an example, the results of a previous study showed that low hairy root growth, biomass, and ginsenoside content were obtained after the experiments using a bioreactor containing normal MS nutrient medium, indicating optimal mineral-element ratio adjustments were required for increasing hairy root growth and biomass. In addition, by exploiting numerous strategies, further enhancements in the accumulation of secondary compounds at both small- and large-scale levels can be gained (Mishra and Ranjan, 2008; Shahzad et al., 2017).

The techniques using hairy root cultures for the production of secondary compounds detected in non-transformed roots were determined (Jabran et al., 2015). The qualifications of hairy root culture techniques for the production of secondary compounds are known as allowing strong secondary compound production through consecutive generations; having genetic stability required for stable productivity; showing hairy root plagiotropism; having exponential growth as biomass increases that occurs as an effect of lateral root formation and results in exponential enlargement in the number of elongation formations, and offering genetic manipulation possibility via transformation to increase biosynthetic capacity. These specifications of the culture techniques were built considerable interest both as a basic study tool and as a source of secondary metabolites (Sathyanarayana and Mathews, 2007).

Hairy root cultures show greater genetic stability than plant suspension cultures. Hairy root plant cultures can be successfully obtained by using different strategies. One of which depends upon the infection occurring between suitable plant explants and *A. rhizogenes* that recognizes a special chemical (acetosyringone) exuded by susceptible wounded plant cells and attaches to them (Gul et al., 2018). The infection of explant with the bacteria causes to arise the development of hairy roots at the site of infection. This approach relies on *A. rhizogenes* mediated continuous hairy root culture together with random gain-of-function mutagenesis (Kayser and Wim, 2006). Later, genetically modified and/or transgenic hairy root cultures are developed, being a great achievement in the system (Mehrotra et al., 2010). A number of research using genetic engineering technology have been realized to improve the production of different kinds of pharmaceutically valuable plant-based bioactive metabolites (Grzegorczyk-Karolak et al., 2018; Mehrotra et al., 2018; Sahai and Sinha, 2022).

Biotechnological applications for the production of secondary metabolites using hairy root cultures of plants have been reported. Followings can be given as examples of secondary compounds produced from hairy roots: podophyllotoxin and related aryltretralin-lignans in *Linum flavum*, (Mikac et al., 2021); curcumin and curcumin monoglucoside in *Atropa belladonna* (Singh et al., 2021b); feruloyl-glucoside in *Turbinicarpus lophophoroides* (Solis-Castañeda et al., 2020); sapogenins (stigmasterol and hecogenin) in *Chlorophytum borivilianum* (Bathoju et al., 2017); alkaloids including eburenine, quebrachamine, fluorocarpamine, pleiocarpamine, tubotaiwine, tetrahydroalstonine and ajmalicine in *Rhazya stricta* (Akhgari et al., 2019); cryptotanshinone and tanshinone in *Perovskia abrotanoides* (Ebrahimi et al., 2017); tropane alkaloids, atropine (hyosciamine) and scopolamine (hyoscine) in *Atropa komarovii* (Banihashemi et al., 2017); flavonoids (rutin, quercetin, isorhamnetin, and isoliquiritigenin) in *Isatis tinctoria* (Jiao et al., 2018); caffeic acid, prolithospermic acid, salvianolic acid J, rosmarinic acid hexoside (I) and (II), salvianolic acid E, methyl rosmarinate, salvianolic acid F (or isomer I and II) in *Salvia viridis* (Grzegorczyk-Karolak et al., 2018); triterpenoids in *Centella asiatica* (Baek et al., 2022) and taxol in *Taxus baccata sub* sp. *wallichiana* (Sahai and Sinha, 2022).

### 4.2 Bioreactors

**Figure 7:**
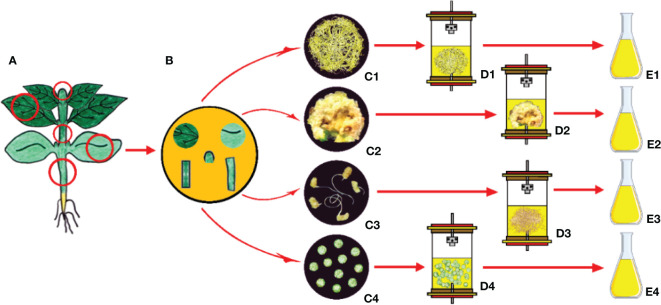
Using bioreactors for secondary metabolite production. **(A)** Main plant and explant sources, **(B)** Isolation of explants from different plant parts, obtaining (C1) Hairy roots, (C2) Callus, (C3) Adventitious roots and (C4) Protoplasts, obtaining secondary metabolites in bioreactors using (D1) Hairy root, (D2) Calllus, (D3) Adventitious roots and (D4) Protoplast cultures (E1-4) Isolation of secondary metabolites from different sources (Copyrighted illustration from Prof. Ozyigit).

As a source of secondary compounds, plants are utilized for the production of pharmaceuticals, flavors, fragrances, coloring agents, food additives, and agrochemicals (**Figure 7**) (Wang et al., 2017). The productions using plants occur regardless of growth parameters and the productions are not in large quantities as well as their occurrences in different tissues of the same plant are at different rates (Atanasov et al., 2015). The roles of most of these compounds in plants are related to defense and as being the leading alternative, the plant cell/organ culture developed has been given the paving of the way for resulting in advancing of bioreactors for plant cell/organ culture (Srikantan and Srivastava, 2018). However, a number of problems observed as cell productivity below desirable level, slow-growing rate, having genetic instability in high-producing cell lines, poor control in cellular differentiation, and failure in maintaining photoautotrophic growth interfere with performance regarding the practices of plant cell cultures (Sajc et al., 2000; Dörnenburg, 2008).

As an efficient way to produce active biomolecules *in vitro*, the process used in plant culture depends on factors such as optimization and engineering that affect plant cells what is needed to scale up large bioreactor volumes (Srikantan and Srivastava, 2018). The cultivation strategy involving using bioreactors for plant cell and hairy root cultures is adopted for providing low shear stress, proper mixing, a suitable support system for organ cultures, and ease in scale-up (Honda et al., 2001). To increase biomass and achieve maximum productivity in plant cell suspensions, bioreactors are designed with sterile conditions and approved batch consistency. Regulatory approval systems compatible with bioreactors allow for more successful and faster operations. (Fischer et al., 2012). The bioreactor system offers some key advantages such as allowing cultures to grow rapidly, promoting the bulk transfer of nutrients and gases, promising to scale up the process and save labor costs. Moreover, constant micro-environmental conditions and a high degree of automation of the cultivation process for a liquid medium can be provided by using the bioreactor systems (Yancheva et al., 2019; Sidal, 2022). Similar to microbial and mammalian cell cultures grown in bioreactors that have fulfilled the requirements set by the FDA and EMEA over the past 20 years, plant cell cultures grown in bioreactors show advantages for the production of therapeutics (Huang and McDonald, 2012). Bioreactor configuration for plant cell cultures shows dependence on aerobic conditions with low shear and good mixing. Due to having bigger cell sizes and forming aggregates or organs in comparison with microbial cultures, the operation of bioreactor for cell suspension cultures or hairy root cultures at constant intervals is difficult in terms of the sampling of biomass (Vaghari et al., 2017; Srikantan and Srivastava, 2018).

Different bioreactor configurations have been developed to provide integrative ability with the biological systems to have efficient growth and better product yield (Sharma and Shahzad, 2013). Bioreactors show modifications, including the addition of mist spray, temporary immersion, having mesh or basket according to the cultivation process being performed for the organized plant structures such as hairy roots, somatic embryos, and micropropagation of plantlets (Paek et al., 2005; Srivastava and Srivastava, 2012). Plant cell bioreactors are designed depending on operative capacity which could be mechanically, hydraulically, pneumatically driven, immobilized, or perfusion types (Eibl and Eibl, 2009; Srikantan and Srivastava, 2018). Physical damage (wounding) and shear stress are the factors causing negative effects on the cultivations of hairy roots and callus in bioreactors, of which hairy roots are more sensitive compared to callus; therefore, the usages of low-shear impellers and external support (stainless steel plate or styrofoam mesh) are essential. Due to excessive branching of hairy roots causing self-immobilization by forming an interlocked matrix in the bioreactor, the biochemical mass transfer of nutrients and oxygen becomes limited (Eibl et al., 2018; Srikantan and Srivastava, 2018, Angulo-Bejarano et al., 2019).

Large-scale cultivations of plant cell-based products such as taxol, shikonin, and taliglucerase alfa in bioreactors have been carried out using plant cell suspension cultures (Hidalgo et al., 2018). A modified impeller bioreactor to reduce cell damage from the hydrodynamic stress on cell suspension that cells are found in a homogeneous state is used for cultivation. Bioreactor types with various configurations, including stirred tank, airlift, and bubble column with minor modifications have been commonly employed to grow plant cell suspension cultures. Cell aggregation, foaming, and cell aggregation are issues that cause problems in bioreactors used for plant cell suspension cultures. These difficulties can be overcome by using a suitable low shear impeller and implementing efficient aeration (Huang and McDonald, 2012; Srikantan and Srivastava, 2018).

As discussed above, there are several ways of improving the production yields of secondary compounds via using plant cell cultures or suspensions. They could be by application of biotic or abiotic elicitors; addition of a precursor leading to improvement in the desired compound; modifying the metabolic carbon flux that leads to promoting the expression pathway of the target compound; generating new genotypes by genetic manipulation using genetic engineering or protoplast fusion; treatment with mutagens causing rise the variability already existing in living cells; and using root cultures (Neumann et al., 2009; Bhaskar et al., 2021). For obtaining high productivity, yield, and concentration, a plant cell/tissue-based bioprocess can be designed by taking into consideration of following important parameters. These are the selection of cell lines giving high yields, media optimization, and strategy for optimal bioreactor operation (Srivastava and Srivastava, 2007; Srikantan and Srivastava, 2018).

As examples of recent studies using bioreactors, the followings can be given: phenolic acids, flavonoids and dibenzocyclooctadiene lignans in *Schisandra chinensis* (Szopa et al., 2018; Szopa et al., 2019); verbascoside, baicalin, wogonoside, luteolin, luteolin-7-glucoside in *Scutellaria alpine* (Grzegorczyk-Karolak et al., 2017); flavonoids in *Gynura procumbens* (Pramita et al., 2018); isoflavonoids in *Pueraria tuberosa* (Kanthaliya et al., 2019); essential oils, p-cymene, geranyl acetate, δ-cadinene, shyobuone, methyl everninate, alloaromadendrene, ledene oxide (II) in *Ledum palustre* (Jesionek et al., 2018); phenylethanoid glycosides (verbascoside,isoverbascoside) and aucubin in *Castilleja tenuiflora* (Cortes-Morales et al., 2018); thapsigargin in *Thapsia garganica* (Lopez et al., 2018); rosmarinic acid and phenolics in *Salvia nemorosa* (Heydari et al., 2020); flavonoids (Quercetin, Kaempferide, Epicatechin gallate, quercetin-3-o-glucose, Kaempferol-3-rutinoside) in *Orostachys cartilaginous* (Hao et al., 2020); antifungal saponins SC-2 and SC-3 in *Solanum chrysotrichum* (Salazar-Magallón and Huerta de la Peña, 2020); six phenolic acids [rosmarinic acid, methyl rosmarinate cafeic acid hexoside, cafeic acid, salvianolic acid F (I) and salvianolic acid F (II)] and four phenylethanoids (verbascoside, leucosceptoside, isoverbascoside and martynoside) in *Salvia viridis* (Grzegorczyk-Karolak et al., 2022); and some phenolic acids, flavonoids (diosmin, catechin, rutin, and myricetin), a stilbenoid (resver- atrol) and phenylethanoid glycosides (acteoside and echinacoside) in *Scrophularia striata* (Ahmadi-Sakha et al., 2022).

## 5 Other *in vitro* applications for obtaining secondary metabolytes

Photoautotrophic micro-propagation refers to sucrose-free propagation in culture media, in which carbohydrate accumulation in tissues grown under *in vitro* conditions and subsequent growth occur entirely depending upon photosynthesis and the presence of inorganic nutrients (Kozai, 2010; Gago et al., 2022). The use of photoautotrophic cultures is proven to be useful way of investigating various aspects of photosynthesis, source-sink regulation, nitrogen metabolism, production of secondary metabolites, and defense response subjects (Segečová et al., 2019). *In vitro* photo-autotrophy can be promoted by removing carbohydrates from the culture medium along with increasing gas exchange in the culture vessel (Xiao et al., 2011; Nguyen et al., 2020; Clapa et al., 2022). As shown by previous works, photoautotrophic micropropagation was applicable approach for a number of plant species to be produced by, as in case of photoautotrophic micro-propagation of *Nicotiana tabacum, Lycopersicon esculentum, Solanum tuberosum*, and *Glycine max*. However, now there is intense demand to establish photoautotrophic micropropagation of economically important crop plants (Roitsch and Sinha, 2002). Iarema et al. (2012) described the influence of the photoautotrophic system on increasing the production of secondary metabolites. Photoautotrophic system presents new prospects in terms of increasing commercial production in 20E levels for *Pfaffia glomerata* as well as providing to conduct basic studies aiming at elucidate the biosynthetic pathway of phytoecdisteroids in plants. The presence of an increasing number of photoautotrophic cultures of different economically important species brings into being the basis for secondary metabolite applications (Fazili et al., 2022).

Finding alternative sources of α-tocopherol by using sunflower species (*Helianthus* sp.) is having a priority for researchers working in this field. It was targeted to increase the level of α-tocopherol by various experiments performed under *in vitro* conditions, such as the addition of homogentisic acid as a biosynthetic precursor, in tissues (e.g. hypocotyls, stems, leaves) of different sunflower species and *in vitro* cultures (Caretto et al., 2004). Gala et al. (2005) showed that the volume of α-tocopherol production can be enlarged by adding jasmonic acid to the culture medium. In a following study performed by Fachechi et al. (2007), it was shown that an increase was achieved in α-tocopherol production by the reduction of the sucrose content via following of adding photomixotrophic to the culture medium. 

Photomixotrophic is a suitable micropropagation approach which includes living organisms that are capable of utilizing sugar-containing medium in terms of having energy source. The degree of dependence is related with the sugar concentration found the medium, the presence of feeding with CO_2_, as well as with a higher photosynthetic photon flux (Emara et al., 2018), The cells grown under photomixotrophic conditions had plastids having photosynthetic activity in increased number in comparison with the cells grown under heterotrophic conditions. However, increased sugar as carbon and energy source utilization by photomixotrophic or photoheterotrophic metabolism is occurred in contrast to photoautotrophic metabolism (Madigan et al., 2003).

As well, sunflower cells cultured with photomixotrophically in comparison with sunflower cells cultured with heterotrophically gave result having more chloroplast in a certain extent as well as an increase in the gene expression of the tocopherol biosynthetic enzyme geranylgeranylpyrophosphate synthase (Fachechi et al., 2007). Plastids in higher plants are known to be the sole sites for the biosynthesis of α-tocopherol (Lichtenthaler et al., 1981) and particularly as in chloroplasts of photosynthetic tissues (Munné-Bosch and Alegre, 2002). Therefore, the α-tocopherol production occurred is related with light exposure given (Geipel et al., 2014).

Hence, using immobilized plant cells as practical way of establishing opportunity for realizing of enhancements in industrial production of secondary metabolites will be widely utilized in future. Research and development are important in the field of the immobilization of plant cells, of which has an important place among the potential benefits being gained by using plant cell cultures (Vitta et al., 2021). Among them, the followings can be mentioned: being prolonged viability of cells in the stationary (and producing) phase; supporting sustaining of biomass over an extended time period; making downstream processing easier (if products are secreted); induction of differentiation shown to be related with enhanced secondary metabolism; alleviating of contamination risk; lessening of shear sensitivity; being increased secondary metabolite secretion, under some circumstances; and lowering of fluid viscosity magnitude in terms of clearing up mixing and aeration problems in cell suspension (Vitta et al., 2021; Krasteva et al., 2021).

Calcium alginate having excellent physical characteristics for use as a cell immobilization agent which is the most common one was exploited in the productions of paclitaxel (Bentebibel et al., 2005; Ochoa-Villarreal et al., 2016), vanillin, ajmalicine and capsaicin (Rao and Ravishankar, 2002; Bentebibel et al., 2005). The application of immobilized plant cells may constitute a significant opportunity to enhance the future industrial production of secondary products. Considering of designing of the culture conditions in optimum level for the production of secondary metabolites and using of several approaches for manipulating the synthesis of these phyto-constituents can be brought into reality by exploiting of approaches such as cell line selection, elicitation, and precursor feeding (Gaosheng and Jingming, 2012).

## 6 Conclusion and future perspectives

Tissue culture techniques are one of the application areas for plant biotechnology, providing extraction of valuable plant metabolites under restrained conditions. Production of secondary metabolites using plant cell and tissue cultures have distinct advantages compared to classical methods. These advantages can be summarized as: the ability of producing the relevant metabolites under controlled conditions without being affected by environmental factors; the culture conditions being optimized in order to increase the production of secondary metabolites; by taking into account the supply-demand balances, sufficient production being provided when necessary, and thus, regularly controlling the market; a quick production without any political pressure; acquisition of disease-free and harmless plant material; and *in vitro* culturability of any plant, whether of having tropical or subtropical originality. 

Secondary compounds being found in the groups of alkaloids, flavonoids, and terpenoids can exhibit a variety of biological activities related with therapeutic use, and some can also be effective in the prevention of various types of diseases. In addition to pharmaceutical industry, their applications find place in paint manufacturing, food processing, cosmetic field, and agricultural management. Therefore, the production of them in large volumes is an important issue. Large-scale production of secondary metabolites can be accomplished using post-harvest isolations from plants grown for specific purposes or using production technology (phytofermentations) in *in vitro* cultures. The combination of these strategies with synthetic biology approaches not only helps in showing the interaction natures between drugs and pathologies but also enhances the efficiencies in the development of new products within related industries.

By discovery of new secondary metabolites and the applications of new analytical methods of the chemical industry for large-scale biotechnological production will provide gains in the area. Therefore, this study aims to demonstrate the importance of secondary compounds produced by plants and to emphasize their evolution in research studies/technologies. Also, information about tissue culture based biotechnological applications is provided to cover in this study. Here, our intent is to give a leading prospect for the related industries in terms of providing sufficient quantity and quality production of these compounds using tissue culture based biotechnological applications. 

## Author contributions

IO: Validation, writing - review & editing, supervision. ID: Conceptualization, investigation. AH-O, BY, AE, IY: Original draft, writing, editing. EC: Investigation. YK: Editing and writing. All authors contributed to the article and approved the submitted version.
